# Evaluating the variety of *GNAS* inactivation disorders and their clinical manifestations in 11 Chinese children

**DOI:** 10.1186/s12902-022-00941-8

**Published:** 2022-03-16

**Authors:** Guoying Chang, Qun Li, Niu Li, Guoqiang Li, Juan Li, Yu Ding, Xiaodong Huang, Yongnian Shen, Jian Wang, Xiumin Wang

**Affiliations:** 1 Department of Endocrinology and Metabolism, Shanghai Children’s Medical Center, School of Medicine, Shanghai Jiao Tong University, 1678 Dongfang Road, Shanghai, 200127 China; 2 Department of Medical Genetics and Molecular Diagnostics Laboratory, Shanghai Children’s Medical Center, School of Medicine, Shanghai Jiao Tong University, 1678 Dongfang Road, Shanghai, 200127 China

**Keywords:** *GNAS*, Pseudohypoparathyroidism, Pseudopseudohypoparathyroidism, Progressive osseous heteroplasia, Albright’s hereditary osteodystrophy

## Abstract

**Background:**

The *GNAS* gene on chromosome 20q13.3, encodes the alpha-subunit of the stimulatory G protein, which is expressed in most tissues and regulated through reciprocal genomic imprinting. Disorders of *GNAS* inactivation produce several different clinical phenotypes including pseudohypoparathyroidism (PHP), pseudopseudohypoparathyroidism (PPHP), progressive osseous heteroplasia (POH), and osteoma cutis (OC). The clinical and biochemical characteristics overlap of PHP subtypes and other related disorders presents challenges for differential diagnosis.

**Methods:**

We enrolled a total of 11 Chinese children with PHP in our study and analyzed their clinical characteristics, laboratory results, and genetic mutations.

**Results:**

Among these 11 patients, nine of them (9/11) presented with resistance to parathyroid hormone (PTH); and nine (9/11) presented with an Albright′s hereditary osteodystrophy (AHO) phenotype. *GNAS* abnormalities were detected in all 11 patients, including nine cases with *GNAS* gene variations and two cases with *GNAS* methylation defects. These *GNAS* variations included an intronic mutation (c.212 + 3_212 + 6delAAGT), three missense mutations (c.314C > T, c.308 T > C, c.1123G > T), two deletion mutations (c.565_568delGACT*2, c.74delA), and two splicing mutations (c.721 + 1G > A, c.432 + 1G > A). Three of these mutations, namely, c.314C > T, c.1123G > T, and c.721 + 1G > A, were found to be novel. This data was then used to assign a *GNAS* subtype to each of these patients with six cases diagnosed as PHP1a, two cases as PHP1b, one as PPHP, and two as POH.

**Conclusions:**

Evaluating patients with PTH resistance and AHO phenotype improved the genetic diagnosis of *GNAS* mutations significantly. In addition, our results suggest that when *GNAS* gene sequencing is negative, *GNAS* methylation study should be performed. Early genetic detection is required for the differential diagnosis of *GNAS* disorders and is critical to the clinician’s ability to distinguish between heterotopic ossification in the POH and AHO phenotype.

## Background

The *GNAS* gene encodes the alpha-subunit of the stimulatory G protein (Gsα) as well as four other transcripts. This gene exhibits parent-specific methylation in its promoters and some transcripts. *GNAS* also experiences a tissue-specific reduction in the expression of the paternal *GNAS* gene in some tissues including the proximal renal tubules, pituitary gland, thyroid, and gonads [1]. Several hormones are regulated via the signal transduction pathway downstream of Gsα and cyclic adenosine monophosphate (cAMP) [2]. Inactivating mutations in exons 1–13 of the *GNAS* gene can result in the expression of a defective Gsα protein, which can cause Albright’s hereditary osteodystrophy (AHO) that presents as tissue resistance to several hormones and distinct skeletal and developmental defects [3].

Pseudohypoparathyroidism (PHP) consists of a heterogeneous group of disorders that exhibit one common feature, namely, resistance to the actions of the parathyroid hormone (PTH). These disorders are roughly classified into PHP types 1 and 2. PHP type 1 is then further classified into three subtypes, namely, 1a, 1b, and 1c based on their clinical presentation, *GNAS* variant, and various laboratory findings, including their response to exogenous PTH infusion and Gsα activity. When heterozygous mutations are located in the maternal allele of *GNAS* exons 1–13, patients exhibited multiple hormone resistance, which act via the Gs-coupled receptors such as parathyroid hormone (PTH), thyroid-stimulating hormone (TSH), gonadotropins, growth hormone-releasing hormone (GHRH), in addition to the more traditional AHO features. This disorder is defined as PHP1a [1]. In contrast, PHP1b is caused by epigenetic abnormalities within differentially methylated regions (DMRs) of *GNAS* which may affect several transcripts, including Gsα, XLas, NESP55, 1A, and antisense (AS) [4]. PHP1b is characterized by resistance to PTH and other hormones. However, these patients do not exhibit AHO phenotypes [1]. PHP1c is caused by receptor coupling defects in the same Gsα and cAMP signal transduction pathway as PHP1a and presents with the same clinical features and laboratory findings [5], but most patients in this group do not present with any of the known pathogenic mutations of the Gsα encoding exons of the *GNAS* gene. Previous studies provided evidence that an overlap exists between the clinical features of different PHP subtypes [6].

Patients more frequently exhibit the AHO phenotype, without hormone resistance, when the same *GNAS* mutations are found on the paternal allele. These conditions are often referred to as pseudopseudohypoparathyroidism (PPHP) [7]. The AHO phenotype includes several physical characteristics, including brachydactyly, stocky build, short stature, obesity, round face, ectopic ossifications, and intellectual disability [1]. These features can also occur in both PHP and PPHP with variable intensity. Gsα haploinsufficiency is thought to play a primary role in the development of the AHO phenotype [2].

Progressive osseous heteroplasia (POH) is another ultra-rare genetic disease caused by heterozygous inactivating mutations in *GNAS* exons 1–13 and is characterized by cutaneous ossifications that proceed into the subcutaneous and deep connective tissues, including muscles, tendons, and ligaments [8]. POH is not however associated with either hormone resistance or the AHO phenotype.

This study evaluated the clinical and genetic characteristics of 11 Chinese children with clinical presentations consistent with *GNAS* spectrum disorders. Our evaluation was largely consistent with previous reports, and of the 11 mutations identified in this study, three were shown to be novel. Because of the overlap between the clinical and biochemical features of different PHP subtypes, accurate diagnosis requires a careful molecular and epigenetic analysis of *GNAS*. Furthermore, two patients were affected by PHP1a and PPHP and were treated with growth hormone (GH) therapy, which provide new data for GH therapy in PHP patients without GH deficiency.

## Methods

### Patients and Clinal data collection

A total of 11 cases of PHP were identified and enrolled in our study at the Shanghai Children's Medical Center, Shanghai Jiaotong University School of Medicine, between July 2017 and May 2021. The documented medical history of each patient including the status of their birth, growth, development, past illness, phenotypes, and family history were preserved for use in this study. Both routine and specific biochemical tests, including electrolyte and 25-hydroxy vitamin D, were performed on each patient. Their PTH, thyroid hormones, insulin-like growth factor-1(IGF-1), and adrenocorticotropic hormone (ACTH) indices were also evaluated. Each patient was also subject to x-ray examination and Cranial MRI or CT, with the exception of patient 2 and patient 5.

Ethical approval for this study was obtained from the ethics committee of the Shanghai Children's Medical Center and informed consent for genetic analysis and the collection of clinical data were obtained from the participant’s healthcare proxies.

### Genetic analysis

Genomic DNA was extracted from the peripheral blood samples collected from each of these 11 patients and their parents using a QIAamp Blood DNA Mini Kit® (Qiagen GmbH, Hilden, Germany). Targeted next-generation sequencing and data analysis were then performed as described in our previous study [9]. All suspected variants were confirmed by Sanger sequencing and validated by parental testing. Manual classification of these variants was then completed using the method recommended by the American College of Medical Genetics and Genomics (ACMG) [10]. Potential pathogenicity of novel missense variants was determined using three in silico prediction methods, including Mutation Taster (http://www.mutationtaster.org), Sorting Tolerant from Intolerant (SIFT; http://sift.jcvi.org/), and Polymorphism Phenotyping v2 (PolyPhen-2; http://genetics.bwh.harvard.edu/pph2/).

Certain samples that were negative for G*NAS* mutation when evaluated by Next generation sequencing (NGS) were then subjected to *GNAS* methylation detection. This methylation analysis was performed using methylation-specific multiplex ligation-dependent probe amplification (MS-MLPA) and the ME031A kit (MRCHolland, Amsterdam, The Netherlands) as per the manufacturer’s instructions. MS-MLPA data was then analyzed using Coffalyser software (MRC‐Holland, the Netherlands).

## Results

### Clinical description and laboratory results for each of the 11 patients in this study

**Fig. 1:**
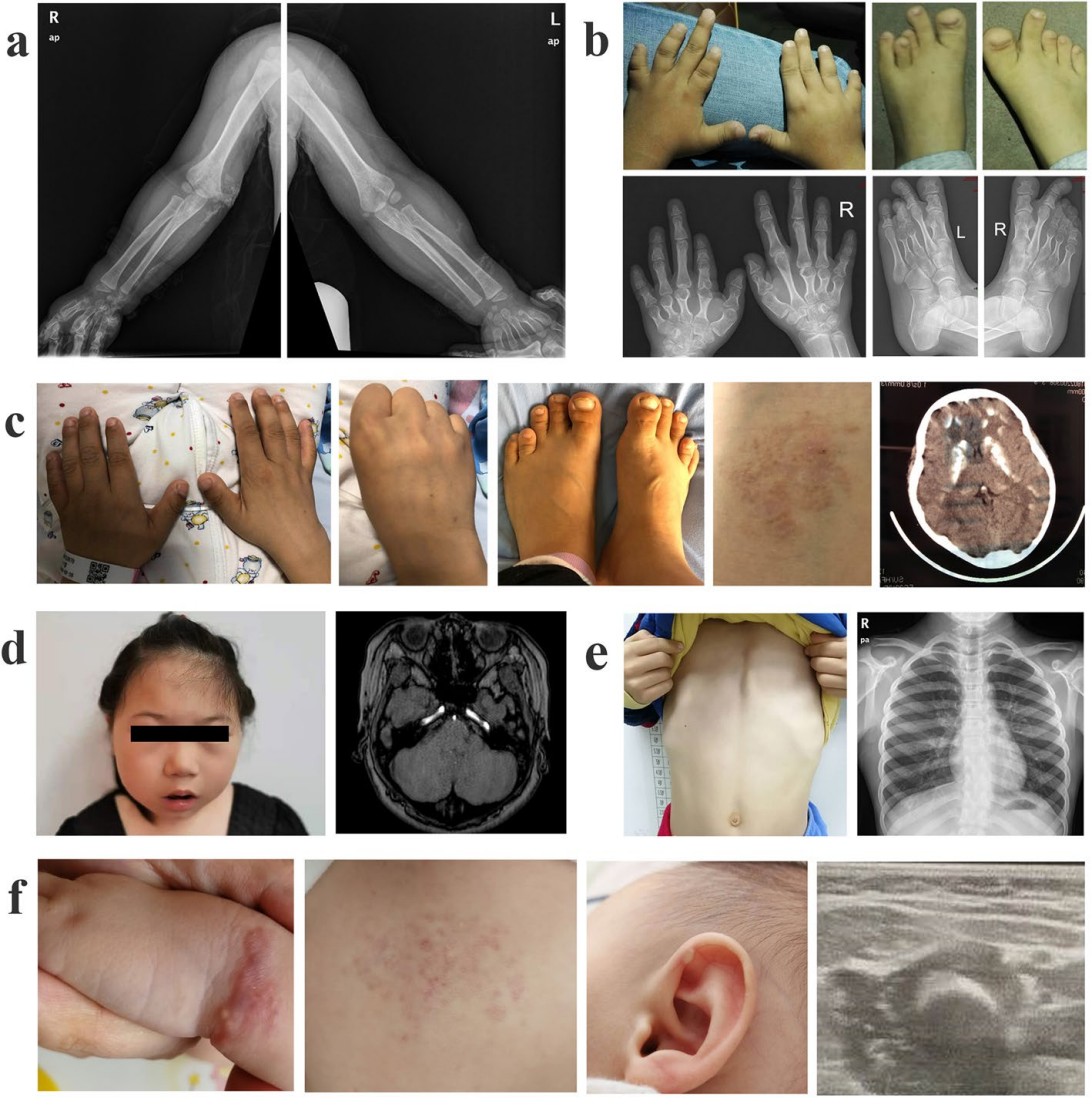
The clinical characteristics of patients with pseudohypoparathyroidism. **a** High density shadows in the subcutaneous soft tissue of both the wrist and right elbow were observed in patient 1.**b** Brachydactyly of the hand and feet digits was presented in patient 2. Shortening of the metacarpals and metatarsals was revealed by X-ray, involving F2-5 on the left, F3-5 on right, and T3-4 on both sides. **c** Brachydactyly of the hands and feet was noted in patient 3. Radiographs of patient 3 revealed shortening of the metacarpals and metatarsals, particularly F1-5 and T4 on both sides. Patient 3 also presented with subcutaneous calcifications across their back, and cranial CT revealed multiple bilateral calcifications involving the cerebral hemisphere. **d** Patient 6 presented with a rounded face, and her head MRI revealed basal ganglia calcification. **e** Patient 9 experienced deformity of thorax, and her chest radiograph revealed scoliosis. **f** Patient 10 suffered from subcutaneous calcifications in the left thigh with developing nodes in the left wrist, both shanks, the back, the abdomen, the auricula, and the inframandibular region. Radiograph revealed high density shadows in both lower limbs

We analyzed the phenotype and genotype of 11 Chinese children with PHP from 11 different families, with no consanguineous marriages. There were six males and five females in our cohort with a male to female ratio of 1.2:1. The age at presentation ranged from neonates to 13 years old, and the age at diagnosis ranged from 6 months to 14 years (the average being 6.5 ± 5.2 years). Among the 11 patients, three cases were referred to our clinic due to heterotopic ossification (P1, P10, P11), four cases for recurrent seizures (P3, P4, P7, P8), and five cases for reduced stature (P2, P4, P5, P6, P9). P6 received growth hormone treatment due to a reduced growth rate, and P9 was previously diagnosed as having idiopathic short stature (ISS) in a local hospital and was also given recombinant human growth hormone (rhGH) therapy. P3 presented with a familial history of AHO-like features. However, patient P3’s mother had no hormone resistance, and was diagnosed as PPHP. These patients’ clinical features are summarized in Table1 and Fig. 1.

**Table 1:** Clinical features of 11 patients with pseudohypoparathyroidism

Patient	Sub type	Gender	Age of onset	At diagnosis				Initial presentation	AHO phenotypes	Family history
				**Age**	**Height(cm)**	**Weight(kg)**	**BMI**			
P1	PHP1a	Female	1.5 y	2.5 y	91 (0.5SD)	20 (4.9SD)	24.2	heterotopic ossification	HO, RF, OB, SB	Positive
P2	PHP1a	Female	3 y	11.8 y	137.8 (-2.2SD)	36.8 (-0.6SD)	19.4	growth retardation	SS, BR, RF	Negative
P3	PHP1a	Male	11 y	11.5 y	133 (-2.2SD)	36.5 (-0.5SD)	20.6	recurrent seizures	SS, BR, MR, HO, RF, SB	Positive
P4	PHP1a	Male	0.5 y	0.75 y	69 (-1.4SD)	8.3 (-1SD)	17.4	recurrent seizures	MR, RF, SB	Negative
P5	PHP1a	Female	8 y	9 y	120.5 (-2.4SD)	23.6 (-1.1SD)	15.3	growth retardation	SS, BR, RF	Negative
P6	PHP1a	Female	3 y	7.8 y	116.8 (-2.7)	30.9 (1.9SD)	22.6	growth retardation	SS, BR, MR, RF, OB, SB	Negative
P7	PHP1b	Male	13.6 y	14 y	157 (-1.2SD)	44.2 (-0.9SD)	17.9	recurrent seizures	NO	Negative
P8	PHP1b	Female	2.3 y	3.5 y	102 (0.7SD)	17 (1.5SD)	16.3	recurrent seizures	NO	Negative
P9	PPHP	Male	3 y	10.3 y	129.5 (-2.1SD)	24.2 (1.8SD)	14.4	growth retardation	SS, BR, HO, OB, SB	Negative
P10	POH	Male	newborn	0.8 y	67 (-2.7SD)	6.8 (-2.8SD)	/	heterotopic ossification	HO,	Negative
P11	POH	Male	newborn	0.5 y	65 (-1.4SD)	6.7 (-1.8SD)	/	heterotopic ossification	HO	Negative

*SS* Short stature, *BR* Brachydactyly, *MR* Mental retardation, *HO* Heterotopic ossification, *RF* Round face, *SB* Stocky build, *OB* Obesity

A total of 54.5% (six cases) of our cohort were diagnosed with PHP1a, 18.2% (2 cases) with PHP1b, 9% (one case) with PPHP, and 18.2% (two cases) with POH. The clinical and laboratory data for each of the 11 cases are summarized in Table 1. All six cases (P1–P6) of PHP1a exhibited features of AHO (shown in Fig. 1). Laboratory tests revealed PTH resistance in all six patients with three cases (P3, P4, P5) presenting with hypocalcemia, five (P1, P3, P4, P5, P6) with TSH resistance, and two cases (P1, P4) with mildly elevated plasma ACTH. Regular X-rays of P1 revealed multiple ossifications in her left upper limb (Fig. 1a), and cranial CT identified multiple bilateral calcifications of the cerebral hemisphere in P3 and P6 (Fig. 1c, 1d).

The two PHP1b cases (P7, P8) presented with recurrent seizures due to hypocalcemia; neither exhibited any symptoms of AHO, presenting with only PTH resistance. Cranial CT of P7 revealed multiple high-density changes, while P9, who was diagnosed with PPHP, demonstrated a mild AHO phenotype, including short stature, brachydactyly and deformity of thorax (Fig. 1e). Because of short stature, P9 was subject to GH stimulation testing which revealed that their GH peak was normal (10.9 µg/µL, 4.7 µg/µL). Moreover, GH treatment has no evident effect. The two POH patients (P10, P11) presented with heterotopic ossification, involving both skin and muscle (Fig. 1f) and demonstrated no hormone resistance. A summary of these laboratory and imaging results is recorded in Table 2.

**Table2:** Laboratory, imaging findings and molecular analysis of 11 patients with PHP

Patient	Sub type	Calcium (mmol/L)	Phosphorus (mmol/L)	PTH (pg/ml)	TSH (uIU/ml)	ACTH (pg/mL)	IGF-1 (ng/ml)	Hormonal resistance	Imaging finding	GNAS abnormality	Inheritance
P1	PHP1a	2.7	1.9	309	12.0	29.5	114	PTH, TSH	Normal	c.212 + 3_212 + 6delAAGT	maternal
P2	PHP1a	2.3	1.7	124.3	4.7	NA	486	PTH	NA	c.314C > T (p.T105I)	De novo
P3	PHP1a	1.3	3.2	172	8.8	12.1	378	PTH, TSH	calcification	c.565_568delGACT (p.D189Mfs*14)	maternal
P4	PHP1a	1.8	2.9	110.3	6.8	34.9	NA	PTH, TSH	Extracerebral space enlargement	c.1123G > T(p.V375L)	maternal
P5	PHP1a	2.1	2.4	728.8	7.2	NA	128	PTH, TSH	NA	c.565_568delGACT (p.D189Mfs*14)	NA
P6	PHP1a	2.3	1.9	282.6	5.8	NA	136	PTH, TSH	calcification	c.432 + 1G > A	De novo
P7	PHP1b	1.5	3.3	506.2	2.4	17.1	199	PTH	Normal	XLas, NESPAS	NA
P8	PHP1b	1.4	2.2	638.4	2.3	10.1	61.2	PTH	Normal	XLas, AS	NA
P9	PPHP	2.5	1.4	41.9	2.1	8.9	161	NO	Normal	c.308 T > C(p.I103T)	De novo
P10	POH	2.4	1.6	50.1	2.2	10.6	NA	NO	Normal	c.721 + 1G > A	De novo
P11	POH	2.5	2.0	42.6	1.4	10.3	NA	NO	Normal	c.74delA, p.K25Rfs*33	De novo

*NA* Not available

### Identification of *GNAS* gene abnormalities

Of the 11 PHP patients in this study, nine presented with variations in the *GNAS* gene and two with *GNAS* methylation defects (Table 2). The *GNAS* gene variations included an intronic mutation (c.212 + 3_212 + 6 delAAGT), three missense mutations (c.314 C > T, c.308 T > C, and c.1123G > T), two deletion mutations (c.565_568delGACT and c.74delA), and two splicing mutations (c.721 + 1G > A and c.432 + 1G > A). Three of these mutations were found to be novel, namely, c.314C > T, c.1123G > T, and c.721 + 1G > A. No mutation of exons 1–13 of *GNAS* were identified in P7 and P8. However, we did detect a loss of methylation in the XLas and NESPAS regions of this gene in P7, and a loss of methylation in the XLas and AS region of this gene in P8.

## Discussion

All of the patients recruited to this study presented with clinical features related to *GNAS* inactivation. We classified these patients into different disease types based on their clinical and molecular evaluations and performed genotype–phenotype correlation analysis. This revealed that patients with PTH resistance, such as P3, may present with hyperphosphatemia, hypocalcemia, and muscle weakness. Despite the presence of PTH resistance in patients 1 and 2, some cases might have elevated PTH levels with normal serum calcium and phosphate levels [11]. There was no resistance to the effects of PTH in the bone or ascending tubules of these patients that could explain this outcome [12]. Other subtypes of PHP could be distinguished from PHP1a based on these features. Furthermore, subclinical hypothyroidism was a hallmark of PHP1a [13]. Both patients, P1 and P3, experienced elevated thyroid stimulating hormone (TSH) levels with normal free thyroxine 4 (FT4) levels, suggesting that both of these patients were suffering from subclinical hypothyroidism. Patients P4, P5, and P6 presented with increased TSH and decreased FT4, which indicated TSH resistance, and all three received L-Thyroxine therapy.

The AHO phenotype, which is characterized by both skeletal and developmental defects, is a group of physical characteristics with variable expressivities. A clinical review showed that there were two different periods characterized by: 1) round face, rapid weight gain, subclinical hypothyroidism, and subcutaneous calcifications in toddlers, and 2) moderate intellectual disability, brachydactyly, afebrile seizures (hypocalcemia), short stature, and TSH resistance in older children [12]. The presentation of our patients were consistent with the above clinical studies. In addition, brachydactyly and heterotopic ossifications have been thought to be the most distinctive feature of the AHO phenotype [13]. Moreover, we observed strabismus in patient 3 and 9; this symptom has been reported in other studies and may represent an additional AHO-related feature [14].

PHP1b is characterized by PTH resistance, hypocalcemia, and hyperphosphatemia, without the addition of AHO-like symptoms. PHP1b is caused by epigenetic changes of the DMR within *GNAS*. Approximately 80% of PHP1b patients present with a sporadic mutation, and only 20% are autosomal dominant (AD) maternally inherited [15]. Both patients P7 and P8 experienced some form of hypocalcemia at 14 and 2 years old, respectively. These patients had been evaluated at the local hospital using NGS, but the results of these evaluations were negative. Given this, we went on to perform *GNAS* methylation detection, and the diagnosis of PHP1b was finally confirmed. This data suggests that patients who present with both PTH resistance and hypocalcemia and without AHO phenotypes, should be routinely evaluated by *GNAS* methylation detection as the first line of testing.

With the exception of those with PHP1b, a large proportion of PHP patients experience short stature into adulthood. This is likely a result of their GHRH resistance, with 50% – 80% of patients with PHP1a presenting with some degree of GH deficiency [16, 17]. The efficacy of rhGH treatment in patients with PHP1a was first studied in 2010, with the evaluation of changes in height velocity in eight prepubertal patients with PHP1A who had GH deficiency following rhGH therapy [18]. P6, who had PHP1a, experienced an increase in their growth rate in response to GH treatment, but P9, who had PPHP and was initially diagnosed with ISS, demonstrated no response to their rhGH therapy. The first international Consensus Statement for PHP recommends that patients with GH deficiencies should be treated with rhGH [19], but its use in patients without GH deficiency needs further evaluation.

Heterotopic ossification in POH can be distinguished from AHO by the presence of progressive ossification moving from subcutaneous tissues into deep connective tissues, as shown in patients P10 and P11. Heterotopic ossification can result in the ankylosis of affected joints and lead to severe disability; follow-up is needed to monitor the progression of this ossification. POH should also be distinguished from other genetic conditions causing heterotopic ossification such as fibro dysplasia ossificans progressive (FOP). These patients did not present with the congenital malformations of the large toes and pre-osseous tumor-like inflammation or “flare-ups” associated with FOP, which aided in their differential diagnosis [20].

The first mutation in *GNAS* was reported in 1990 [21] with numerous other mutations described since then. These include missense mutations, splicing substitutions, small/cross deletions or insertions, regulatory substitutions, small indels, and complex rearrangements. A small 4-bp deletion at codons 189–190 in exon 7 (c.565_568delGACT) of *GNAS* has been confirmed to be a mutational hot spot [22], and this hot spot was present in two of our patients (P3 and P5). We also identified a heterozygous 4-bp deletion mutation (c.212 + 3_212 + 6delAAGT) in P1, which is predicted to disrupt the highly conserved 5' splice site sequence in intron 2 and activate a cryptic splice site located 28 bp downstream of intron 2 [23]. P2 had a novel missense variant, c.314C > T(p.Thr105Ile), in exon 5. Exon 5 of the *GNAS* gene encodes the adenylate cyclase activation domain of Gsα, in which amino acids 70 to 140 have been reported to be important for functionality in activating adenylyl cyclase [24]. P4 had a novel nucleotide exchange c.1123G > T in exon 12, leading to a Valine to Leucine substitution (p.Val375Leu). Furthermore, multi-bioinformatics in silico software predicted that these variants have deleterious effects, and both variants were classified as likely pathogenic using the ACMG guidelines. Patient 10 had a novel splicing variant (c.721 + 1 G > A) in exon 9, which occurs within the + 1 splice site (PSV1) and was de novo (PS2), indicating that this variant is also pathogenic (PSV1 + PS2 + PM2 + PP4). P6 presented with a c.432 + 1G > A mutation; however, the same splicing mutation was reported in a boy with recurrent medulloblastoma [25], indicating that our patient had a risk of developing tumors, suggesting that she will need careful monitoring and that her rhGH treatment should be more conservative.

Our study was a retrospective study from a single center, and the sample size was small. Large sample studies are needed to broaden the range of our results. Moreover, the effect of rhGH therapy in PPHP or other types of PHP, should be further investigated.

## Conclusions

We completed a phenotypic and molecular assessment of 11 patients with PHP, which can be difficult to diagnose as its clinical phenotype is highly variable, and Gsα activity is not routinely assessed or available. Therefore, NGS and methylation detection of *GNAS* serve as reliable methods of confirming PHP diagnosis. We demonstrate that patients who present with PTH resistance and no AHO phenotype should be evaluated for changes in *GNAS* methylation to aid in differential diagnosis. In addition, our data supports the conclusion that it is necessary for clinicians to distinguish between the heterotopic ossification associated with POH and the AHO phenotype. This study not only expands our understanding of the phenotypic spectrum of *GNAS* mutations, but also deepens our understanding of the clinical phenotype associated with alterations in the *GNAS* gene.

## Data Availability

All the data during this study are included in this published article.

## Abbreviations

PHP: Pseudohypoparathyroidism
PPHP: Pseudopseudohypoparathyroidism
POH: Progressive osseous heteroplasia
OC: Osteoma cutis
PTH: Parathyroid hormone
AHO: Albright′s hereditary osteodystrophy
Gsα: Alpha-subunit of the stimulatory G protein
cAMP: Cyclic adenosine monophosphate
TSH: Thyroid-stimulating hormone
GHRH: Growth hormone-releasing hormone
DMRs: Differentially methylated regions
AS: Antisense
GH: Growth hormone
IGF-1: Insulin-like growth factor-1
ACTH: Adrenocorticotropic hormone
NGS: Next generation sequencing
MS-MLPA: Methylation-specific multiplex ligation-dependent probe amplification
ISS: Idiopathic short stature
rhGH: Recombinant human growth hormone
TSH: Thyroid stimulating hormone
FT4: Free thyroxine 4
AD: Autosomal dominant
FOP: Fibro dysplasia ossificans progressive
